# Prediction of fat-free mass and fat mass from bioimpedance spectroscopy and anthropometry: a validation study in 7- to 9-year-old Kuwaiti children

**DOI:** 10.1017/S1368980025000503

**Published:** 2025-04-21

**Authors:** Tareq Al-Ati, Jonathan Wells, Leigh C Ward

**Affiliations:** 1 Food and Nutrition Program, Environment and Life Sciences Research Center, Kuwait Institute for Scientific Research, P.O. Box 24885, Safat 13109, Kuwait; 2 Childhood Nutrition Research Centre, UCL Institute of Child Health, London, UK; 3 School of Chemistry and Molecular Biosciences, The University of Queensland, St Lucia, Brisbane, QLD 4072, Australia

**Keywords:** Bioelectrical impedance analysis (BIA), Sum of skin-folds, Percentage body fat, Body composition analysis, Fat-free mass, Total body water, Deuterium dilution, Children, Kuwait

## Abstract

**Objective:**

Childhood obesity is increasing in many countries, including Kuwait. Currently, adiposity is most commonly assessed from simple anthropometric measurements, e.g. height and weight or combined as body mass index (BMI). This is despite these surrogate measurements being poor indices of adiposity. Bioelectrical impedance analysis (BIA) is a popular method for the assessment of body composition providing a measurement of adiposity as absolute fat mass (FM) or FM expressed as a percentage of body weight (%BF). BIA is, however, an indirect predictive method. This study developed a BIA-based prediction equation for body composition assessment in Kuwaiti children and, additionally, a prediction equation for %BF based on sum of skin-fold (SSF) thickness measurements.

**Design:**

A cross-sectional design was used with primary school recruitment.

**Setting:**

School population in Kuwait City; in-clinic assessments.

**Participants:**

158 Kuwaiti children aged 7–9 years. Body composition assessed using bioimpedance spectroscopy and skin-folds with prediction equations generate against deuterium dilution measurement of total body water and fat-free mass (FFM) as reference.

**Results:**

The newly developed and cross-validated BIA equation predicted FFM with minimal bias (< 1%) and acceptable 2 sd limits of agreement (±1·6 kg equivalent to ±10%) improving on the predictive performance of comparable published equations. Similarly, SSF predicted %BF with small bias (0·2 %BF) but relatively wide limits of agreement (±7 %BF).

**Conclusions:**

These new equations are suitable for practical use for nutritional assessment in Kuwaiti children, particularly in epidemiological or public health settings although their applicability in other populations requires further research.

The prevalence of pediatric obesity is increasing in Kuwait^(1)^. The most recent survey (Kuwait Nutrition Surveillance System) in 2018 reports 16·5 % of 5–6 year-old boys and girls are obese rising to 32·8 % of boys and 27·4 % of girls aged 9–10^(2)^. A further 19·7 % of boys and 28·8 % of girls were overweight at age 9–10 years in 2018. The most common way to classify pediatric obesity is body mass index (BMI, body weight in kilograms divided by height in meters squared) according to age and sex. Although BMI is efficient in providing a useful indication of body size and shape, it does not provide a precise measure of body composition or spatial information of body components, for example distribution of body fat or adipose tissue. Furthermore, the relationship between body fat and BMI tends to differ between different ethnic groups. For example, when comparing adult white Europeans, South Asians, Africans and Pacific Islanders, for the same BMI value, Africans have been reported to have the least fat mass (FM), while the South Asians and Pacific Islanders have the highest^(3)^. Another study indicates that in white children of the same age and sex, fat mass can differ by two-fold while BMI remains the same^(4)^. BMI tends to misclassify children with high bone or muscle mass since it does not differentiate between FM and fat-free mass (FFM)^(5)^. Excess body fat is misinterpreted by BMI, accordingly, a more accurate method for assessing body fat is needed.

Obesity is correlated with morbidity and mortality therefore a more reliable and practical method for assessing adiposity is required for a large population^(6)^. Many methods are considered reliable for adiposity assessment, however, not all are appropriate in children or in public health settings. Dual- energy X-ray absorptiometry (DXA) and MRI are inappropriate for a large population because of their high cost^(7)^ while the radiation hazard associated with DXA may preclude its use in children. Deuterium dilution can be used to assess total body water (TBW) which following application of age and sex appropriate hydration factors can estimate FFM. The technique is considered a reference method but is generally not routinely usable owing to cost and the need for specialist analytical laboratory facilities^(8)^. Alternatively, studies have shown, that less costly but reliable methods such as bioelectrical impedance analysis (BIA) and skin-fold thickness (SF) are acceptable for use in children^(9,10)^. These methods are, however, indirect relying upon empirically-derived prediction equations to measure body composition, typically as absolute FFM and FM or %body fat (%BF). Most published equations have been generated for Caucasians and are widely regarded as being ethnicity-specific^(11)^. The Kuwaiti population is relatively homogeneous, being derived from the major population groups of the Arabian Peninsula^(12)^. Thus, the aim of this study was to develop new anthropometric and BIA equations, based on deuterium dilution^(13)^ as the reference method, to assess body composition for Kuwaiti children.

## Materials and methods

The study was a method comparison and validation sub-study as part of a program to establish body composition methodologies at the Kuwait Institute of Scientific Research (KISR). The establishment of the reference deuterium dilution technique has been described previously in the same children as described here^(13)^.

### Participants

A total of 158 7- to 9-year-old children (75 boys and 83 girls) were recruited from primary public schools from Kuwait City, Kuwait. This age group and sample size was chosen to complement a similar study being undertaken under the auspices of the International Atomic Energy Agency (IAEA) in Asian children^(14)^ A non-random purposive sampling approach was used to enrol children encompassing a wide BMI range for each year of age and sex as in^(14)^.

Inclusion criteria were a healthy Kuwaiti child not on medication or has a medical condition that may affect body composition or metabolic rate. The study was conducted in accordance with the guidelines laid down in the Declaration of Helsinki and all procedures involving human participants were approved by the Joint Committee for the Protection of Human Subjects in Research at the Kuwait Institute of Medical Specialization of the Ministry of Health. All children, their parents and teachers were informed about the aims and procedures of the study and a completed written parental consent form was obtained for each child.

### Anthropometric measurements

Participants were received in the health clinic at KISR. Three trained Ministry of Health professionals were responsible for taking all the anthropometric measurements. Each professional was responsible for one anthropometric measurement for all participants to minimize inter-researcher error. Height was measured, with the children in the Frankfort plane, using a portable stadiometer (Holtain, Crymych, Pembs, UK). Body weight was measured using a digital electronic scale (SECA Robusta 813, Advanced Technology Company K.S.C., Hawali, Kuwait), while the participant was barefoot and wearing light clothes. The average of three measurements was used for the final measured value with a maximum allowable difference of 0·1 cm for height and 0·1 kg for weight, measurement resolution for height and weight respectively. BMI classification and criteria were based on WHO BMI-for-age growth chart ^(15)^, using the calculated BMI (weight(kg)/height^2^(m^2^)). Classification of children as normal weight, overweight or obese based upon BMI-for-age z-score has been described previously^(13)^.

Skin-fold thickness was measured with a Holtain skin-fold caliper; measurements were taken while the child was standing and on the right side of the body. An average of three measurement was taken on the four sites (triceps, biceps, subscapular, and suprailiac), with a maximum allowable difference of 0·2 mm.

Waist and hip circumferences were measured using non-stretchable measuring tape; waist circumference (WC) (cm) was measured around the child’s bare abdomen at midpoint between lower rib and iliac crest using and hip circumference (HC) (cm) was measured at widest part around hip. All measurements were obtained in triplicate, to 0·1 cm resolution with 0·5 cm as the maximum allowable difference between the three measurements and the mean values used.

### Bioimpedance spectroscopy measurement

#### Protocol

A tetrapolar electrical bioimpedance spectrometer (Imp SFB7, ImpediMed Limited, Pinkenba, Qld, Australia) was used to measure whole body, wrist-to-ankle impedance (Z, ohm) resistance (R, ohm) and reactance (Xc, ohm). Prior to taking the measurements all metal accessories, socks and shoes were removed. Measurements were taken on the right side of the body while lying supine, EKG-style electrodes (single tab, ImpediMed Limited, Pinkenba, Qld, Australia) were placed 5 cm apart on the hand and foot in the conventional manner as described elsewhere^(16,17)^. Measurements were taken after the participant had been in supine for 10 min during the deuterium dilution equilibration phase. Factors known to influence hydration status and BIA measurements including exercise, eating, drinking were supervised and study took place in an air-conditioned room temperature (22°C within a degree) with participants all measured after an overnight fast as described previously^(13)^. Measurements were taken in duplicate; each measurement taking approximately 1 s.

#### Data analysis

Impedance spectrum data were analysed according to the Cole model^(18)^ using Bioimp software (v4.5.0.0, Impedimed Ltd., Brisbane, Australia). The resistance at 50 kHz (R50) was extracted for generation of a single frequency prediction equation and the resistance at zero (R0) and infinite (Rinf) frequencies were obtained from extrapolation of data from a graphical plot of the Cole model^(18)^ and used to predict TBW and FFM according mixture theory^(19)^ using the body composition module of Bioimp. Additional parameters required were the apparent resistivities of intra- and extracellular water (ICW and ECW respectively), a body proportion factor (K_B_), body density and an hydration fraction (HF) to convert TBW to FFM (TBW/HF). Published body proportion factors are limited to adults (K_B_≅4·3) but may be computed from anthropometric measurements^(20)^. A K_B_ value was estimated using anthropometric data obtained in the present study and from the literature^(21)^. Age and sex appropriate hydration factors were taken from Wells *et al.* ^(22)^ In preliminary analyses the older published factors of Lohman were tested and yielded slightly larger (3 %) TBW values^(23)^. Resistivity coefficients for children have not been published so those used by Moon *et al.* ^(24)^ and Ward *et al.* ^(25)^ were tested. In addition, Moissl’s modification of mixture theory incorporating BMI was assessed^(26)^.

### Total body water measurement by deuterium dilution

Total body water was measured by the deuterium dilution technique following the protocol of the IAEA^(8)^ as described in detail previously^(13)^. Briefly, a baseline urine sample was collected after an overnight fast, followed by administration of a 10 % D_2_O dose based on body weight. Post-dose urine samples were collected 4 h after the intake of the dose. Urine samples were analysed to determine deuterium enrichment using isotope ratio MS (IRMS, Nu Instruments, UK). A 4 % correction for the difference between isotope dilution space and TBW was applied. FFM was calculated from TBW using the same hydration fraction as used for the impedance data, i.e. age and sex appropriate hydration factors according to Wells *et al.* ^(22)^.

### Data analysis

#### Anthropometric predictions of body composition

Multiple regression was used to predict %BF from skin-fold measurements. To be consistent with published prediction equations the sum of skin-folds (SSF) was log transformed^(27)^. The dependent variable was %BF determined from deuterium dilution, independent (predictor) variables were SSF and sex (coded female = 0, male = 1). Assumptions of normality and constant variance made in multiple regression were checked and met. Multi-collinearity between independent variables was assessed by determining the variance inflation factor (VIF); a value less than 10 being deemed acceptable. The se of the estimate error, calculated as the square root of the mean square error, coefficient of determination (R^2^) values, Akaike information criterion (AIC), Mallow’s Cp and the predicted residual error sum of squares (PRESS) statistic were used to determine goodness of fit of the regression model.

A double cross-validation was performed in which a randomized, sex-stratified 50:50 split of the total sample was carried out (*n* 79 per group). Equations developed in each group were cross-validated in the opposite group. Covariance analysis and comparison of the slopes and intercepts was used to compare the regression models between the two groups and if NS, a single equation from the whole sample was also generated with predicted performance assessed using the LOOCV procedure (Solverstat 2019 R0; software available at https://solverstat.wordpress.com/, last accessed 29th November 2022). The performance of the new prediction equation was compared to published prediction equations for similar populations of children (see online supplementary material, Supplemental Table 1)^(9,28–33)^. For those equations which predict body density^(28,31–33)^, %BF was calculated using the modified Siri equation of Weststrate and Deurenberg^(34)^ as recommended by Reilly *et al.* ^(27)^

#### Bioelectrical impedance predictions of body composition

The measured resistance at 50 kHz (R50) was used to develop prediction equations using stepwise multiple regression analysis. FFM was the dependent variable and the predictor variables examined were weight, age, sex (male = 1, female = 0), and resistance index (RI) based on height (height^2^/resistance). Assumptions of normality and constant variance and multi-collinearity were assessed as used for anthropometric prediction. Cross-validation of the best performing predictor was undertaken using the same split-group procedure as for anthropometric predictions. Single-frequency predictions of FFM were compared with both published prediction equations for similar populations and those determined using mixture theory modelling as described above.

### Statistical analysis

Statistical analyses were performed using MedCalc v19.8 for Windows (MedCalc Software, Broekstraat 52, B-9030 Mariakerke, Belgium). Results are expressed as mean and sd except where otherwise indicated. Differences in participant characteristics between sexes were examined by independent sample *t* test for continuous variables and z-test for proportional variables. Statistical significance was defined as *P* < 0·05. Data were assessed for normality using Kolmogorov-Smirnov test and for outliers using generalized extreme studentized deviate (ESD) procedure at an alpha level of 0·05.

Predictive performance of equations, either anthropometric or impedance-derived, was assessed using Pearson correlation, paired sample *t* test, Lin’s concordance correlation, Cohen’s D for paired samples, residual sd for random differences (Passing-Bablok regression) and Bland-Altman limits of agreement analysis. Relative ranking of prediction equations was based upon median absolute percentage error (MAPE), two-one-sided *t* test equivalence analysis for paired data and Gower similarity index. The TOST procedure measures the minimum % difference at which the mean values are considered equivalent and were calculated using the Excel spreadsheet provided by Lakens (available at https://files.osf.io/v1/resources/q253c/providers/osfstorage/58455f749ad5a100475fe734?action=download&direct&version=7, last accessed 29th November 2022). The Gower index varies between 0 and 1 where 1 represents identity between measurements. Gower indices were calculated using Gower (v1·1) (available at https://www.pbarrett.net/Gower/Gower.html, last accessed 29th November 2022) Statistical significance was set at *P* < 0·05.

## Results

### Participant characteristics

Physical and reference body composition data of the participants are presented in Table 1. Total participant number was 158 (75 boys and 83 girls) aged 7- to 9-years-old Kuwaiti children. There were no significant differences in any body composition parameter with the exception of %BF between the sexes. A wide range in %BF was observed, varying from 14·2 % to 57·5 % with %body fat being significantly larger (*P* < 0·01) in females than males (means 38·7 % *v*. 35·1 %). BMI classification, based on the WHO growth reference charts^(15)^, showed a bimodal distribution with similar proportions of boys and girls in the normal and obese classes (40–50·7 %) and few (8–16·9 %) classed as overweight. General characteristics of the two sub-groups of participants (prediction generation and validation) are presented in Table 2. There were no significant differences and each group was representative of the whole population.

**Table 1.** Physical and body composition characteristics of participants

Variable	Males (*n* 75)			Females (*n* 83)			*P*-value*
	Mean	sd	Range	Mean	sd	Range	
Age (years)	8·2	0·6	(7·0–9·4)	8·1	0·7	(7·0–9·4)	0·79
Weight (kg)	33·9	10·8	(19·1–65·5)	33·6	11·1	(18·2–76·53	0·88
Height (cm)	129·8	6·4	(113·9–148·8)	129·0	7·4	(112·2–149·5)	0·49
Triceps skinfold (mm)	14·1	6·7	(5·0–31·0)	14·5	5·2	(5·1–25·3)	0·66
Biceps skinfold (mm)	8·50	5·2	(2·5–22·0)	8·7	4·2	(3·0–20·0)	0·83
Supscapular skinfold (mm)	12·5	9·1	(3·4–35·0)	12·5	7·0	(5·0–32·0)	1·00
Suprailiac skinfold (mm)	13·0	9·4	(2·9–34·0)		12·77·5	(2·8–34·07)	0·83
Waist circumference (cm)	63·6	12·9	(48·0–99·0)	62·9	12·2	(44·7–102·0)	0·72
Hip circumference (cm)	74·8	10·9	(59·0–102·0)	75·2	10·7	(58·0–112·0)	0·84
Waist-hip ratio	0·84	0·06	(0·75–0·99)	0·83	0·06	(0·7–1·0)	0·68
BMI (kg/m^2^)	19·8	5·1	(13·6–35·2)	19·8	4·7	(13·5–35·7)	0·98
	*n*	%		*n*	%		
BMI classification							
Normal (%)	38	50·7		36	43·4		
Overweight (%)	6	8		14	16·9		
Obese (%)	31	41·3		33	39·8		
	Mean	sd		Mean	sd		
BMI z-score†	1·60	2·0	(–1·9–6·8)	1·4	1·5	(–1·3–5·3)	0·39
TBW (kg)	16·3	3·0	(11·2–28·1)	15·4	3·4	(10·0–28·3)	0·39
Fat-free mass (kg)	21·1	3·9	(14·5–36·5)	19·9	4·4	(13·0–36·8)	0·39
Fat mass (kg)	12·7	7·2	(2·8–39·7)	13·8	7·4	(4·9–34·1)	0·34
%BF	35·1	9·8	(14·2–55·0)	38·7	7·8	(24·7–57·5)	0·01
FFMI (kg/m^2^)	12·5	1·5	(9·9–17·5)	11·8	1·5	(9·4–17·1)	0·44
FMI (kg/m^2^)	7·4	3·9	(2·1–18·2)	8·0	3·4	(3·3–18·5)	0·44

Abbreviations: BMI = body mass index; TBW = total body water; %BF = percentage body fat. Values are means ± sd, range in parenthesis. *Differences were analyzed with a two-sample *t* test. †WHO growth reference standards^(15)^.

**Table 2.** General characteristics of participants for the split samples

Variable	Group 1 (*n* 79)		Group 2 (*n* 79)		*P*-value*
	%	*n*	%	*n*	
Female, %(*n*)	51·9	41	53·2	42	0·73†
	Mean	sd	Mean	sd	
Age (years)	8·1	0·7	8·2	0·7	0·18
Weight (kg)	33·7	10·6	33·8	11·3	0·97
Height (cm)	129·0	6·8	129·8	7·1	0·51
BMI (kg/m^2^)	19·7	4·9	20·0	4·9	0·77
Impedance (Z, Ω)	738·3	92·5	734·7	80·6	0·79
Resistance (R, Ω)	735·4	92·4	731·7	80·7	0·79
Reactance (Xc, Ω)	64·8	7·1	64·8	7·7	0·95
Height^2^/Resistance (RI, cm^2^/ Ω)	23·2	4·5	23·5	4·4	0·67
TBW (kg)	15·8	3·2	15·8	3·3	0·89
Fat-free mass (kg)	20·5	4·1	20·6	4·3	0·87
Fat mass (kg)	13·3	6·9	13·3	7·7	0·97
%BF (%)	37·2	8·7	36·8	9·3	0·75

Abbreviations: BMI = body mass index. Values are means ± sd. *Differences were analyzed with a two-sample *t* test; †z-test for proportions.

### Anthropometric prediction of body composition

Prediction of %BF fat from log-transformed sum of skin-folds is presented in Table 3. Prediction models were developed for each sub-group for both males and females separately and by including sex as a predictor variable. Relationships between predictor variables and reference %body fat were highly significant (*P*< 0·0001) with strong coefficients of determination (R^2^), > 0·83 except for females alone, R^2^ = 0·76. Prediction error (RMSE) was similar across all models, approximately 3·5 kg.

**Table 3.** Anthropometric predictive models for percentage body fat* in Kuwaiti children aged 6·5–9·6 years

Participant group	Model	Regression coefficient	*P* value	R^2^	RMSE (kg)	Mallow’s	PRESS	Akaike	VIF
	Predictors					C(p)		information criterion	
Group 1	LogSSF +	32·36	0·0001	0·834	3·697	3	1064	204·4	1
	Sex+	−2·862	0·0001						
	Intercept	−14·07							
Group 2	LogSSF +	35·005	0·0001	0·846	3·578	3	1104	209·7	1
	Sex+	−1·903	0·0001						
	Intercept	−18·696							
All participants	LogSSF +	33·578	0·0001	0·839	3·624	3	2114	409·8	1
	Sex+	−2·437	0·0001						
	Intercept	−16·203							
All females	LogSSF +	32·532	0·0001	0·757	3·857	2	na	226·1	na
	Intercept	−14·4916	0·0001						
All males	LogSSF +	34·2637	0·0001	0·885	3·36	2	na	183·8	na
	Intercept	−19·7375	0·0001						

Abbreviations: SSF = sum of 4 skin-folds (biceps, triceps, sub-scapular, suprailiac); RMSE = root mean square error, PRESS = predicted error sum of squares: VIF = variance inflation factor; na = not applicable to single variable regression;* reference method, deuterium dilution.

Cross-validation of the generated prediction equation (including sex as a predictor variable) was excellent with minimal bias (±< 0·3 kg) as indicated by high correlation (> 0·91), high % similarity (TOST < 2·5 % similarity), high Gower index and low MAPE (< 6·5 %) (top panel, Table 4). Pearson and concordance correlations between predicted and reference %BF values were virtually identical indicating no systematic differences. Overall prediction error was small (2·5 kg) with 1·96 sd limits of agreement between methods of ±7·1 kg.

**Table 4.** Anthropometric prediction of percentage body fat* in Kuwait children aged 6·5–9·6 years

Prediction method	Validation group	Sex	Mean (%BF)	sd	Bias* (%BF)	sd	Limits of agreement (%)	*r*_*p*_	*r*_*c*_	Cohen’s d effect size for paired data	RSD (%BF)	MAPE (%)	TOST (%)	Gower index
Split-group cross-validation														
Reference %BFD_2_O	Group 2	Both	36·8	9·3										
Prediction Group 1 (Table 3)	Group 2	Both	36·7	8·1	0·1	0·8	−7·2–7·4	0·918	0·909	0·03	2·5	6·2	2·2	0·943
Reference %BFD_2_O	Group 1	Both	37·2	8·7										
Prediction Group 2 (Table 3)	Group 1	Both	37·4	8·5	−0·2	0·7	−7·2–6·9	0·911	0·911	0·05	2·6	6·5	2·4	0·932
Comparison with published predictors (all participants)														
Reference %BFD_2_O	All	M	35·1	9·8										
	All	F	38·7	7·8										
This study (LOOCV)	All	M	35·1	9·0	0·0	3·3	−7·5–7·5	0·941	0·938	0·30	2·3	5·1	1·8	0·927
	All	F	38·7	7·0	0·0	3·8	−7·5–7·5	0·870	0·865	0·92	2·7	6·4	1·8	0·901
Durnin and Rahman^(28)^	All	M	14·5	8·5	20·6	3·4	13·9–27·3	0·941	0·262	6·54	2·9	61·3	60·7	0·588
	All	F	20·0	6·3	18·8	3·8	11·6–25·6	0·870	0·186	6·36	3·1	48·1	50·1	0·625
Slaughter *et al.* ^(9)^	All	M	22·1	6·5	12·1	3·8	4·6–19·7	0·934	0·546	4·30	3·9	40·9	39·5	0·757
	All	F	22·9	10·8	16·6	4·0	9·1–23·7	0·856	0·226	3·12	3·3	43·2	43·7	0·668
Johnston *et al.* ^(32)^	All	M	12·8	9·2	22·3	3·3	15·8–28·8	0·941	0·248	6·83	3·1	68·7	65·4	0·554
	All	F	16·6	6·2	22·1	3·9	14·9–28·9	0·870	0·142	6·15	3·1	56·9	60·0	0·558
Wendel *et al.* ^(29)^	All	M	30·3	7·5	4·8	4·0	−3·1–12·6	0·928	0·778	1·45	2·8	13·6	15·9	0·791
	All	F	34·3	6·1	4·4	4·1	−3·0–11·6	0·849	0·687	1·14	3·3	11·7	13·3	0·898
Brook^(33)^	All	M	18·2	10·5	16·9	3·5	9·9–23·4	0·941	0·394	4·84	3·6	50·8	50·1	0·663
	All	F	18·2	10·3	20·5	5·2	10·3–30·4	0·870	0·236	4·40	5·1	53·8	55·3	0·589
Deurenberg *et al.* ^(31)^	All	M	20·8	7·6	14·2	3·7	6·9–21·6	0·940	0·389	4·75	3·3	41·5	42·8	0·715
	All	F	24·0	6·5	14·7	3·8	7·7–21·4	0·869	0·273	4·01	3·2	37·3	39·9	0·705
Alkutbe *et al.*	All	M	25·6 7·5		9·5 3·5		1·1–18·3	0·896	0·530	2·39	2·7	27·0	30·5	0·707
	All	F	29·1 7·4		9·4 4·4		2·5–16·3	0·889	0·493	2·68	2·5	25·0	26·4	0·660

*Reference (from deuterium dilution) – predicted. Abbreviations: RSD = residual standard deviation; MAPE = median absolute percentage error; TOST = two one-sided *t* tests.

Comparison of predictive performance of the new equation with comparable and commonly used published equations is present in the lower panel, Table 4. Data are presented for all participants stratified by sex. Generally, published equations, although providing highly correlated data, performed poorly at predicting body composition both at the population level, biases ranging from 4·4 to 22·3 kg, and in individuals with limits of agreement up to ±21·9 kg Table 4, see online supplementary material, Supplemental Figure 1). The best performing published equation was that of Wendel *et al.* ^(29)^

### Prediction of total body water from bioelectrical impedance measurements

Four different models were explored for predicting TBW from impedance measurements using: resistance index (RI, height^2^/R at 50 kHz); weight alone; RI and weight and RI, weight and sex. Each model was developed separately in cross-validation groups 1 and 2 (Table 5). Generally, all potential predictor variables were highly significant (*P*< 0·001) with high coefficients of determination (R^2^) being observed (> 0·87). Sex was the weakest predictor variable being NS in Group 2 but significant in group 1. Prediction error was around 1 kg for all models. Overall, the final prediction model included all three variables with an R^2^ of 0·934 and prediction error of 0·84 kg.

**Table 5.** Predictive models for total body water in Kuwaiti children aged 6·5–9·6 years

Model	Predictors	Regression coefficient	P value	R^2^	RMSE (kg)	Mallow’s	PRESS	Akaike	VIF
						C(p)		information criterion	
Group 1 (*n* 79; 41 F, 38M)									
Model 1	RI +	0·6593	0·0001	0·886	1·082	2	95·2	14·5	na
	Intercept	0·4915							
Model 2	Weight +	0·2821	0·0001	0·872	1·148	2	110·7	23·8	na
	Intercept	6·2631							
Model 3	RI +	0·3715	0·0001	0·939	0·796	3	54·5	33	4·2
	Weight +	0·1422	0·0001						4·2
	Intercept	2·3668							
Model 4	RI +	0·3417	0·0001	0·943	0·777	4	52·7	36	4·7
	Weight +	0·1521	0·0001						4·5
	Sex +	0·411	0·0295						1·1
	Intercept	2·5269							
Group 2 (*n* 79; 42 F, 37M)									
Model 1	RI +	0·6912	0·0001	0·872	1·186	2	117	28·9	na
	Intercept	−0·3894							
Model 2	Weight +	0·2694	0·0001	0·855	1·261	2	128·5	36·1	na
	Intercept	6·4724							
Model 3	RI +	0·3934	0·0001	0·923	0·922	3	71·6	9·84	4·1
	Weight +	0·1346	0·0001						4·1
	Intercept	2·0565							
Model 4	RI +	0·37	0·0001	0·926	0·909	4	71·1	10·8	4·5
	Weight +	0·1433	0·0001						4·4
	Sex +	0·3648	0·0926						1·1
	Intercept	2·1431							
All participants (*n* 158; 83 F, 75 M)									
Model 4	RI +	0·3549	0·0001	0·934	0·836	4	115·3	52·5	4·5
	Weight +	0·1479	0·0001						4·4
	Sex +	0·3873	0·0063						1·1
	Intercept	2·3525							

RI = resistance index (height^2^/R, cm^2^/ Ω); Weight (kg); Sex (M = 1, F = 0); Height (cm), Xc (reactance, Ω). R^2^ = coefficient of determination. Abbreviations: RMSE = root mean square error; PRESS = predicted residual error sum of squares; VIF = variance inflation factor; na = not applicable to single variable regression.

The results of cross validation are presented in the upper panel, Table 6. Bias was minimal <±0·5 kg with small limits of agreement (±1·6 kg).

**Table 6.** Comparison of predictive impedance-based equations for total body water in Kuwaiti children aged 6·5–9·6 years

Prediction	Validation group	Mean (kg)	sd	Bias* (kg)	sd	Limits of agreement (kg)	Cohen’s d effect size for paired data	*r*_*p*_	*r*_*c*_	RSD (kg)	MAPE (%)	TOST (%)	Gower index
Split-group cross-validation													
Reference IRMS-TBW (kg)	Group 2	15·8	3·3										
Prediction model 4 Group 1 (Table 5)	Group 2	15·9	3·1	−0·04	0·9	−1·8–1·7	0·012	0·962	0·961	0·63	2·5	1·4	0·971
Reference IRMS-TBW (kg)	Group 1	15·8	3·2										
Prediction model 4 Group 2 (Table 5)	Group 1	15·7	3·1	0·05	0·6	−1·4–1·5	0·015	0·971	0·971	0·54	2·8	1·6	0·968
Comparison with published predictors (all participants)													
TBWD_2_O (kg)	All	15·8	3·2										
Prediction model 4, Table 5 (LOOCV)	All	15·8	3·1	0·0	0·8	−1·6–1·6	0·00	0·966	0·966	0·58	2·8	0·7	0·967
Moissl *et al.* ^(26)^	All	16·2	3·0^†^	−0·38	1·0	−2·3–1·6	0·40	0·952	0·943	0·70	4·3	3·3	0·960
Moon *et al.* ^(24)^ (K_B_ = 4·3)	All	16·6	3·8^†^	−0·79	1·2	−3·0–1·4	1·02	0·963	0·926	0·67	5·5	6·6	0·947
Ward *et al.* ^(25)^ K_B_ = 4·3	All	16·1	3·7^†^	−0·3	1·0	−2·3–1·6	1·30	0·966	0·951	0·64	4·3	8·3	0·932
Ward *et al.* ^(21,25)^ K_B_ = Personal	All	15·2	3·8	0·61	1·1	−1·4–2·8	0·14	0·965	0·939	0·65	6·0	1·9	0·947
Clasey *et al.* ^(35)^	All	17·1	3·2^†^	−1·31	0·9	−3·2–0·5	1·37	0·956	0·881	0·69	9·0	9·0	0·928
Rush *et al.* ^(36)^	All	18·1	4·0^†^	−2·35	1·2	−4·7–0·0	2·68	0·964	0·779	0·67	14·8	14·8	0·897
Jemaa *et al.* ^(37)^	All	17·4	3·8^†^	−1·57	1·2	−4·0–0·9	1·57	0·949	0·851	0·79	9·7	9·7	0·918
De Lorenzo *et al.* ^(38)^	All	17·8	3·6^†^	−2·01	1·0	−4·0 –0·0	2·33	0·964	0·817	0·65	12·8	12·8	0·898
El Harchaoui *et al.* ^(39)^	All	14·9	3·7^†^	0·88	1·0	−1·1 – –2·9	1·05	0·964	0·926	0·66	7·0	7·0	0·945
Horlick *et al.* ^(40)^	All	16·5	3·5^†^	−0·72	1·0	−2·6–1·1	0·81	0·964	0·939	0·64	5·2	5·2	0·952

*Reference-predicted. Abbreviations: *r*_*p*_ = Pearson correlation coefficient; *r*_*c*_ = concordance correlation coefficient; RSD = residual standard deviation MAPE = median absolute percentage error; TOST = two one-sided *t* tests. ^†^ *P*< 0·001 (paired *t* test).

Comparison with published equations are presented in the lower panel of Table 6 and online supplementary material, Supplemental Figure 2. Comparison was performed against bioimpedance spectroscopic (BIS) prediction approaches (methods of Moissl^(26)^, Moon^(24)^ and Ward^(21,25)^) and empirically-derived single-frequency (50 kHz) prediction equations (Clasey^(35)^, Rush^(36)^, Jemmaa^(37)^, De Lorenzo^(38)^, El Harchaoui^(39)^ and Horlick^(40)^). The prediction equation generated in this study performed the best closely followed by the BIS methods of Ward using personalised K_B_ values and Moissl. The best performing single frequency predictors were those of El Harchaoui and Horlick. Limits of agreement for these predictions were broadly similar (ranging from ±2·0 to ±2·2 kg).

### Prediction of body composition (FM and FFM) from bioelectrical impedance and skin-fold measurements

**Figure 1:**
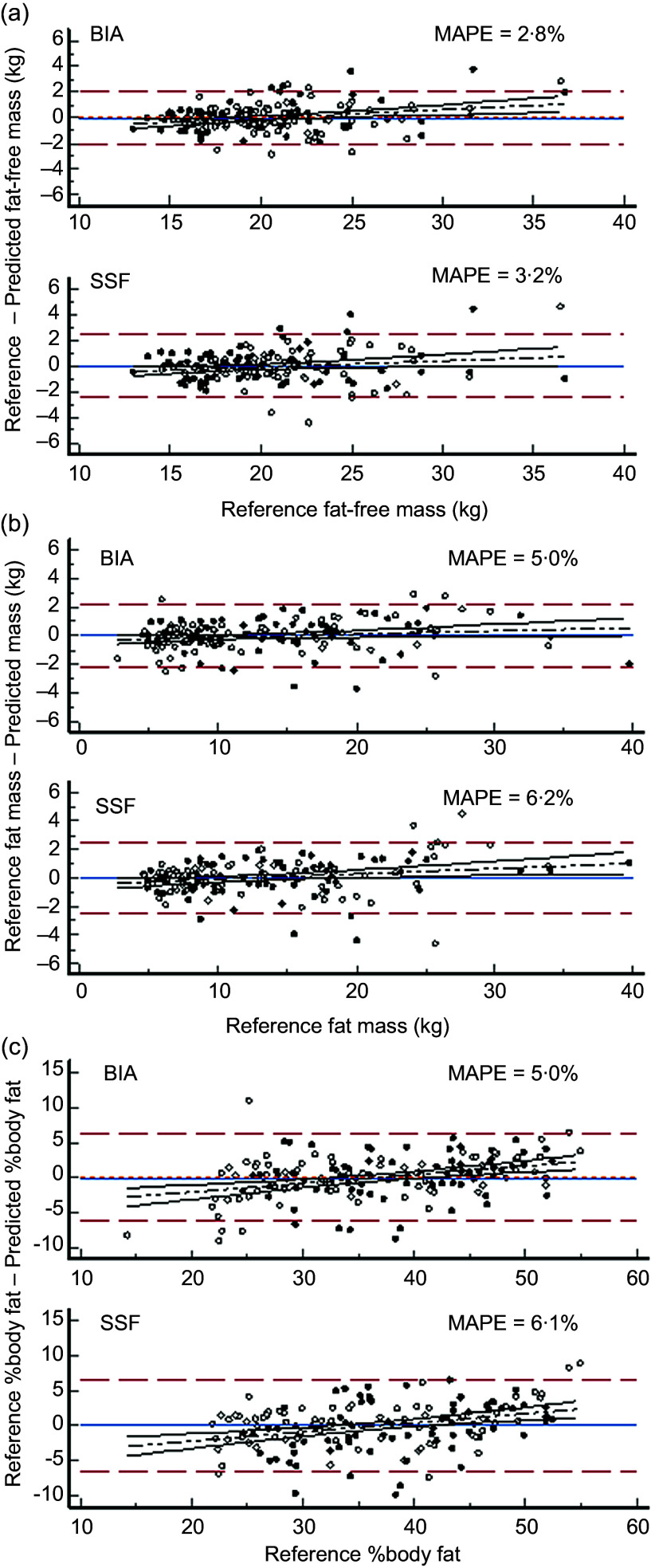
Comparison of body composition predicted by skin-fold-based anthropometric and impedance-based algorithms compared to the reference method of deuterium dilution. Panel A: Fat-free mass (kg). Panel B: Fat mass (kg). Panel C: Percentage body fat (%BF). Key 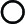: Female participants. 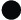: Male participants. 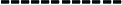: Limits of agreement (± 1·96 sd). 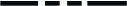
Regression line. 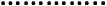: Bias.

Total body water volumes for all participants obtained using the best performing BIA-based predictor equation (Model 4, Table 5) were transformed to FFM values using age-appropriate hydration fractions. Fat mass was determined by difference with body weight and %BF calculated. Similarly FFM and FM were calculated from the derived skin-fold equation for %body fat (Table 4). Predicted FFM, FM and %BF values were compared to reference values calculated from deuterium dilution-determined TBW using limits of agreement analysis and are presented in Figure 1.

Mean differences in FFM were close to zero (MAPE approximately 3 %) for both the BIA-based predictions and the SSF-based predictions with limits of agreement similar (±9·9 % for BIA and 11 % for SSF) to those seen in the split-group analysis, Tables 4 and 6 for BIA and SSF predictions respectively, (Figure 1(a)). A slight but significant (*P* < 0·05) proportional bias was also observed. Impedance-based prediction was slightly better than SSF-based prediction, MAPE = 2·8 % compared to 3·2 % respectively. Figure 1(b) and (c) present similar analyses for FM and %BF. Conversely, SSF predicted FM and %BF slightly better than BIA with limits of agreement of approximately ±17·5 % compared to ±21 % although MAPE was slightly smaller for BIA (5 %) than for SSF (6·1 %).

## Discussion

Childhood obesity in Kuwait is currently estimated to have a prevalence of around 30 %^(2)^ and is a recognised risk factor for CVD and metabolic syndrome^(41)^ in this population. However, most estimates of adiposity and body composition in general are based on either simple height and weight measures or BMI^(42,43)^ despite the acknowledged inadequacies of these measurements for this purpose^(44)^. The present study has provided a validated BIA prediction equation for body composition for use in Kuwaiti children.

BIA technology is popular for estimating body composition as a practical alternative to expensive and technically complex methods such as DXA suitable for use in epidemiological and public health settings. The BIA method is based upon a two-compartment model of the body (body mass = fat mass + fat-free mass) and is an indirect method since it does not measure body composition *per se* but predicts this using transformation algorithms from the measured electrical resistance of the body. Since transmission of an electrical current through the body occurs via the conductive water containing tissues, electrical resistance is quantitatively related to body water volume by


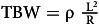
 (1)

where TBW = total body water (L), L = the conductive length (cm), (typically stature is used as a proportional surrogate since true conductive length is unknown), R (ohm) is the measured resistance and ρ is the specific resistivity (ohm.cm). Transformation algorithms are typically empirically derived by calibration against a reference body composition method and developed by regression of the RI *(L^2^/R)* against reference TBW volume. Additional potential predictor variables may be included in a multiple regression. In the present study, the accepted reference method of deuterium dilution was used to measure TBW. Multiple regression was used to develop the best-fitting prediction algorithm with the final equation including RI, sex and weight. Although included as a predictor in the final model, the effect size of sex was relatively small and, in the cross-validation, was only a significant predictor in one of the two validation groups. This is not entirely surprising since sex-differences in body composition of children are generally considered to occur in both FFM and, primarily, in FM in post-pubertal children^(10)^. Sex as a predictor variable did, however, improve albeit slightly prediction performance supporting its retention in the final model. Furthermore, the ratio of intra-to extracellular water in TBW is generally higher in females again supporting inclusion of sex as a covariate^(45)^.

Although BIA is most appropriately calibrated against TBW, of more practical interest to nutritionists is body composition in terms of FM and FFM. TBW may be converted to estimates of FFM by assuming an hydration fraction for FFM. Body water volume and as a proportion of FFM is generally held within a tight physiological range with 0·732 being the accepted assumed hydration fraction of FFM in healthy adults^(46)^. Hydration fraction is higher in newborns and infants and gradually approaches adult values around puberty^(46)^. In the present study, age-appropriate hydration fractions were used to convert TBW volumes to FFM and, in accord with the 2-compartment model, provide estimates of FM and %BF. The newly-developed prediction equation provided excellent prediction of FFM at the population level (< 1 % bias), the limits of agreement were relatively wide, ±9·9 % but comparable to or smaller than those observed in other studies and for alternative prediction equations tested in this population. There is no consensus as to what is a clinically acceptable agreement limit, although 10 % is often considered acceptable^(47)^.

Although BIA is most appropriately used to predict FFM, in clinical practice it is more commonly used to predict FM and %BF. Since these are calculated by difference of FFM and body weight, this is becomes a doubly-indirect prediction with the likelihood of increased error due to error propagation. Error may be further compounded as a result of this calculation involving subtracting FFM that is typically proportionally larger than FM from body weight to calculate the much smaller FM. In the present study, for example, mean FFM was approximately 2-fold larger than FM but was up to 6-fold larger for the participant with the lowest %BF (14·2 %). Notably however, SSF which more directly assesses body fat as subcutaneous adipose tissue exhibited smaller limits of agreement than BIA. These observations suggest that, while the differences are small, BIA may be more appropriate to assess FFM and SSF adiposity in this age group.

The newly developed SSF and impedance predictors were compared to a number of published equations. These equations were selected since they are in widespread use or were developed in comparable populations of children. In general published SSF equations markedly underestimated %BF compared to the reference values. The notable exception being predictions obtained with the equation of Wendel *et al.* which although still underestimating were closer to reference values. Wendel *et al.* and others, e.g.^(48)^ also observed underestimation of %BF when predicted by these same published equations. The reasons for this underestimation are unclear. It is possible that equations such as those of Durnin and Rahman^(28)^ or Brook^(33)^ are now over five decades old and were developed in populations of children with body compositions that are not commensurate with those of contemporary children. A further possibility is that SSF-based equations typically predict body density which is then transformed to %BF. The original transformation method, and still in common use, was the Siri equation^(49)^. In the present study, we used the modification recommended by Weststrate and Deurenberg^(34)^. This produces lower estimates of %BF than Siri; for example, mean %BF from Durnin and Rahman using this approach was 17·4 % but was increased to 24·0 % when transformed using the Siri equation. The newly developed prediction equation did not require conversion of body density to %BF since %BF was calculated directly from FM calculated from deuterium dilution-derived FFM. Similarly the best performing Wendel *et al.* equation also did not invoke body density since this equation was derived again from directly measured %Bf using dual-energy X-ray absorptiometry.

Prediction of FFM from impedance measurements was generally acceptable at a population level, the worst performing published equation (Rush *et al.*) overestimated mean FFM by 15 % with most equations providing estimates within 10 % of reference values. Limits of agreement were typically around ±2 kg or ±12 %. The newly developed equation had minimal bias on cross-validation (0·05 kg) and improved limits of agreement ±1·6 kg or ±10 %. Although there is no acknowledged performance standard for predicting body composition, limits of agreement of this magnitude are generally considered as clinically acceptable for clinical use in individuals^(47)^. The generalizability of the new equation is unknown. Empirically-derived prediction equations frequently show population specificity and are not readily transferable between populations. Conversely, bioimpedance spectroscopy approaches for assessing body composition use a biophysical modelling approach to estimating TBW and FFM and are considered to be less susceptible to population specificity since the models use directly values for resistivity (Equation 1)^(19)^. However, it should be recognized that these resistivity values still require empirical determination and, to date, this has only been undertaken in adults^(25)^. Nonetheless, BIS prediction of TBW performed creditably when appropriate corrections for difference in body proportions of children compared to adults was applied^(21)^.

The study has strengths and limitations. A strength of the study was acknowledgement that although BIA assesses directly FFM, %BF is often of greater interest to dietitians and public health nutritionists. Analysis of data was extended to consider predictive performance for FM and %BF. In addition to BIA, the independent method of skin-fold measurements was assessed and shown to provide acceptable estimates of %BF comparable with those from BIA. The current study used a BIS device, which is typically more expensive and technologically more complex than single frequency bioimpedance analysis (SFBIA) devices. The new equation was, however, developed using impedance data collected at 50 kHz, the frequency used by SFBIA devices extending usability to lower resource settings. A limitation of the study is the relatively narrow age range of the participants, 7–9 years. The newly developed equation should be tested for predictive performance across a wider range of ages although good predictive performance of BIS indicates broader applicability of this approach.

### Conclusions

Both a sum of skin-folds prediction equation for %BF and a single frequency BIA prediction equation for estimation of TBW and FFM in Kuwaiti children aged 7–9 years that showed good predictive performances were developed. The minimal mean biases indicate that the equations should be particularly useful in epidemiological research and for providing body composition data when developing public health strategies to combat increasing childhood obesity. The acceptable limits of agreement suggest that the equations should also prove useful when assessing body composition in an individual in a clinical setting.

## Supporting information

Al-Ati et al. supplementary material Al-Ati et al. supplementary material

## Supplementary material

For supplementary material accompanying this paper visit https://doi.org/10.1017/S1368980025000503

## References

[1] Al-Taiar A , Alqaoud N , Ziyab AH et al. (2021) Time trends of overweight and obesity among schoolchildren in Kuwait over a 13-year period (2007–2019): repeated cross-sectional study. Public Health Nutr 24, 5318–5328. 34342262 10.1017/S1368980021003177 PMC10195387

[2] Ministry of Health (MOH) & State of Kuwait (2018) Kuwait Nutrition Surveillance System 2018 Report, available at https://data.worldobesity.org/country/kuwait-115/report-card.pdf (accessed 19 May 2025).

[3] Rush EC , Freitas I & Plank LD (2009) Body size, body composition and fat distribution: comparative analysis of European, Maori, Pacific Island and Asian Indian adults. Br J Nutr 102, 632. 19203416 10.1017/S0007114508207221

[4] Wells J (2000) A Hattori chart analysis of body mass index in infants and children. Int J Obes 24, 325–329. 10.1038/sj.ijo.0801132 10757626

[5] Freedman DS , Wang J , Maynard LM et al. (2005) Relation of BMI to fat and fat-free mass among children and adolescents. Int J Obes 29, 1–8. 10.1038/sj.ijo.0802735 15278104

[6] Lobstein T , Baur L & Uauy R (2004) Obesity in children and young people: a crisis in public health. Obes Rev 5, 4–85. 15096099 10.1111/j.1467-789X.2004.00133.x

[7] Eisenmann JC , Heelan KA & Welk GJ (2004) Assessing body composition among 3- to 8-year-old children: anthropometry, BIA, and DXA. Obes Res 12, 1633–1640. 15536227 10.1038/oby.2004.203

[8] International Atomic Energy Agency (2010) *Introduction to Body Composition Assessment using the Deuterium Dilution Technique with Analysis of Urine Samples by Isotope Ratio Mass Spectrometry. Human Health*. vol. 1. Vienna: Marketing and Sales Unit, Publishing Section International Atomic and Energy Agency.

[9] Slaughter MH , Lohman TG , Boileau RA et al. (1988) Skinfold equations for estimation of body fatness in children and youth. Hum Biol 60, 709–723. 3224965

[10] Weber DR , Leonard MB & Zemel BS (2012) Body composition analysis in the pediatric population. Pediatr Endocrinol Rev 10, 130–139. 23469390 PMC4154503

[11] Nightingale CM , Rudnicka AR , Owen CG et al. (2013) Are ethnic and gender specific equations needed to derive fat free mass from bioelectrical impedance in children of South Asian, Black African-Caribbean and White European origin? Results of the assessment of body composition in children study. PLoS One 8, e76426. 24204625 10.1371/journal.pone.0076426 PMC3799736

[12] Alsmadi O , Thareja G , Alkayal F et al. (2013) Genetic substructure of Kuwaiti population reveals migration history. PLoS One 8, e74913. 24066156 10.1371/journal.pone.0074913 PMC3774671

[13] Al-Ati T , Preston T , Al-Hooti S et al. (2015) Total body water measurement using the 2 H dilution technique for the assessment of body composition of Kuwaiti children. Public Health Nutr 18, 259–263. 26263176 10.1017/S1368980013003534 PMC10271037

[14] Liu A , Byrne NM , Ma G et al. (2011) Validation of bioelectrical impedance analysis for total body water assessment against the deuterium dilution technique in Asian children. Eur J Clin Nutr 65, 1321–1327. 21731041 10.1038/ejcn.2011.122

[15] De Onis M , Onyango AW , Borghi E et al. (2007) Development of a WHO growth reference for school-aged children and adolescents. Bull World Health Organ 85, 660–667. 18026621 10.2471/BLT.07.043497 PMC2636412

[16] Brantlov S , Jødal L , Lange A et al. (2017) Standardisation of bioelectrical impedance analysis for the estimation of body composition in healthy paediatric populations: a systematic review. J Med Eng Technol 41, 460–479. 28585459 10.1080/03091902.2017.1333165

[17] Brantlov S , Ward LC , Jødal L et al. (2017) Critical factors and their impact on bioelectrical impedance analysis in children: a review. J Med Eng Technol 41, 22–35. 27648845 10.1080/03091902.2016.1209590

[18] Cornish BH , Thomas BJ & Ward LC (1993) Improved prediction of extracellular and total body water using impedance loci generated by multiple frequency bioelectrical impedance analysis. Phys Med Biol 38, 337–346. 8451277 10.1088/0031-9155/38/3/001

[19] Matthie JR (2008) Bioimpedance measurements of human body composition: critical analysis and outlook. Expert Rev Med Devices 5, 239–261. 18331184 10.1586/17434440.5.2.239

[20] Matthie J , Zarowitz B , De Lorenzo A et al. (1998) Analytic assessment of the various bioimpedance methods used to estimate body water. J Appl Physiol Bethesda Md 1985 84, 1801–1816. 10.1152/jappl.1998.84.5.1801 9572833

[21] Ward LC , Wells JCK , Lyons-Reid J et al. (2022) Individualized body geometry correction factor (KB) for use when predicting body composition from bioimpedance spectroscopy. Physiol Meas 43, 035006. 10.1088/1361-6579/ac5e83 35294931

[22] Wells JCK , Williams JE , Chomtho S et al. (2010) Pediatric reference data for lean tissue properties: density and hydration from age 5 to 20 years. Am J Clin Nutr 91, 610–618. 20089731 10.3945/ajcn.2009.28428

[23] Lohmann T (1992) Estimating body composition in children and the elderly. In Advanced Body Composition Assessment, pp. 65–77 [ T Lohmann , editor]. Champaign, IL: Human Kinetics Publishers.

[24] Moon JR , Smith AE , Tobkin SE et al. (2009) Total body water changes after an exercise intervention tracked using bioimpedance spectroscopy: a deuterium oxide comparison. Clin Nutr Edinb Scotl 28, 516–525. 10.1016/j.clnu.2009.04.025 19500888

[25] Ward LC , Isenring E , Dyer JM et al. (2015) Resistivity coefficients for body composition analysis using bioimpedance spectroscopy: effects of body dominance and mixture theory algorithm. Physiol Meas 36, 1529–1549. 26034992 10.1088/0967-3334/36/7/1529

[26] Moissl UM , Wabel P , Chamney PW et al. (2006) Body fluid volume determination via body composition spectroscopy in health and disease. Physiol Meas 27, 921–933. 16868355 10.1088/0967-3334/27/9/012

[27] Reilly JJ , Wilson J & Durnin JVGA (1995) Determination of body composition from skinfold thickness: a validation study. Arch Dis Child 73, 305–310. 7492193 10.1136/adc.73.4.305 PMC1511327

[28] Durnin JVGA & Rahaman MM (1967) The assessment of the amount of fat in the human body from measurements of skinfold thickness. Br J Nutr 21, 681–689. 6052883 10.1079/bjn19670070

[29] Wendel D , Weber D , Leonard MB et al. (2017) Body composition estimation using skinfolds in children with and without health conditions affecting growth and body composition. Ann Hum Biol 44, 108–120. 27121656 10.3109/03014460.2016.1168867 PMC5559202

[30] Hlúbik P , Chaloupka J , Opltová L et al. (1998) Non-invasive methods for evaluation of body composition during weight reduction. Sb Lek 99, 265–266. 10358422

[31] Deurenberg P , Pieters JJL & Hautvast JGAJ (1990) The assessment of the body fat percentage by skinfold thickness measurements in childhood and young adolescence. Br J Nutr 63, 293–303. 2334665 10.1079/bjn19900116

[32] Johnston JL , Leong MS , Checkland EG et al. (1988) Body fat assessed from body density and estimated from skinfold thickness in normal children and children with cystic fibrosis. Am J Clin Nutr 48, 1362–1366. 3202085 10.1093/ajcn/48.6.1362

[33] Brook CGD (1971) Determination of body composition of children from skinfold measurements. Arch Dis Child 46, 182–184. 5576028 10.1136/adc.46.246.182 PMC1647464

[34] Weststrate JA & Deurenberg P (1989) Body composition in children: proposal for a method for calculating body fat percentage from total body density or skinfold-thickness measurements. Am J Clin Nutr 50, 1104–1115. 2816795 10.1093/ajcn/50.5.1104

[35] Clasey JL , Bradley KD , Bradley JW et al. (2011) A new BIA equation estimating the body composition of young children. Obesity 19, 1813–1817. 21681223 10.1038/oby.2011.158

[36] Rush EC , Puniani K , Valencia ME et al. (2003) Estimation of body fatness from body mass index and bioelectrical impedance: comparison of New Zealand European, Maori and Pacific Island children. Eur J Clin Nutr 57, 1394–1401. 14576752 10.1038/sj.ejcn.1601701

[37] Ben Jemaa H , Mankaï A , Khlifi S et al. (2019) Development and validation of impedance-based equations for the prediction of total body water and fat-free mass in children aged 8–11 years. Clin Nutr 38, 227–233. 29429643 10.1016/j.clnu.2018.01.028

[38] De Lorenzo A , Pietrobelli A , Faith MS et al. (1998) Predicting fat-free mass in children using bioimpedance analysis. FASEB J 12, 212–215. 10.1007/s00592-003-0069-z 14618476

[39] El Harchaoui I , El Hamdouchi A , Baddou I et al. (2018) Development and validation of bioelectrical impedance analysis equations for prediction total body water and fat-free mass using D 2 O technique in Moroccan children aged between 8 and 11 years old. Eur J Clin Nutr 72, 1663–1672. Springer US. 29391592 10.1038/s41430-018-0093-2

[40] Horlick M , Arpadi SM , Bethel J et al. (2002) Bioelectrical impedance analysis models for prediction of total body water and fat-free mass in healthy and HIV-infected children and adolescents. Am J Clin Nutr 76, 991–999. 12399270 10.1093/ajcn/76.5.991

[41] El-Bayoumy I & Shalaby S (2011) Metabolic syndrome among obese Kuwaiti adolescents (11–17 Years). J Obes Weight Loss Ther 2, 2–5.

[42] Elkum N , Alarouj M , Bennakhi A et al. (2019) The complex etiology of childhood obesity in Arabs is highlighted by a combination of biological and socio-economic factors. Front Public Heal 7, 1–8. 10.3389/fpubh.2019.00072 PMC6455072 31001508

[43] Boodai SA , McColl JH & Reilly JJ (2014) National Adolescent Treatment Trial for Obesity in Kuwait (NATTO): project design and results of a randomised controlled trial of a good practice approach to treatment of adolescent obesity in Kuwait. Trials 15, 234. 24943283 10.1186/1745-6215-15-234 PMC4074381

[44] Vanderwall C , Randall Clark R , Eickhoff J et al. (2017) BMI is a poor predictor of adiposity in young overweight and obese children. BMC Pediatr 17, 4–9. 28577356 10.1186/s12887-017-0891-z PMC5457636

[45] Tagliabue A , Cena H & Deurenberg P (1996) Comparative study of the relationship between multi-frequency impedance and body water compartments in two European populations. Br J Nutr 75, 11–19. 8785181 10.1079/bjn19960106

[46] Hewitt MJ , Going SB , Williams DP et al. (1993) Hydration of the fat-free body mass in children and adults: implications for body composition assessment. Am J Physiol – Endocrinol Metab 265, E88–E95. 10.1152/ajpendo.1993.265.1.E88 8338157

[47] Ward LC (2019) Bioelectrical impedance analysis for body composition assessment: reflections on accuracy, clinical utility, and standardisation. Eur J Clin Nutr 73, 194–199. Springer US. 30297760 10.1038/s41430-018-0335-3

[48] Aguirre CA , Salazar GDCC , Lopez de Romaña DV et al. (2015) Evaluation of simple body composition methods: assessment of validity in prepubertal Chilean children. Eur J Clin Nutr 69, 269–273. 25097002 10.1038/ejcn.2014.144

[49] Siri WE (1956) The gross composition of the body. Adv Biol Med Phys 4, 239–280. 13354513 10.1016/b978-1-4832-3110-5.50011-x

