# Supplementary material for: Prediction of fat-free mass and fat mass from bioimpedance spectroscopy and anthropometry: a validation study in 7- to 9-year-old Kuwaiti children
Source: Public Health Nutr. 2025 Apr 21;28(1):e95. doi: 10.1017/S1368980025000503 (PMC12171910; doi:10.1017/S1368980025000503)
Supplement: Al-Ati et al. supplementary material [file S1368980025000503sup001.docx]

**Prediction of fat-free mass and fat mass from bioimpedance spectroscopy and anthropometry: a validation study in 7- to 9-year-old Kuwaiti children**

**Legends to supplementary figures**

**Supplementary Figure 1**: Body fat percentage (%BF) predicted by different anthropometric algorithms compared to the reference method of deuterium dilution

Data are presented as limits of agreement plots [difference between methods (deuterium-derived reference – named prediction method} plotted against the deuterium reference values].

Key ⭘ Female participants

⚫ Male participants

**------------** Limits of agreement (± 1.96 SD)

Regression line

………….. Bias

**Supplementary Figure 2**: Total body water (L) predicted by different impedance algorithms compared to the reference method of deuterium dilution (D_2_O)

Data are presented as limits of agreement plots [difference between methods (deuterium-derived reference – named prediction method} plotted against the deuterium reference values].

Key ⭘ Female participants

⚫ Male participants

**------------** Limits of agreement (± 1.96 SD)

Regression line

………….. Bias

**Supplementary Figure 1**

**Supplementary Figure 2**

**Supplementary Table 1: Prediction equations for body density and %BF**

| Reference | Sex  (n) | Age (years)  Mean or range | %BF | Ethnicity | Body density^a^  (BD, kgL^-1^) | %BF |
| --- | --- | --- | --- | --- | --- | --- |
| Durnin and Rahaman ^(26)^ | Male  (48) | 12.7 to 15.7 | 15.9  (5.4 to 34.7) | Caucasian  UK | 1.1533 - 0.0643 * log SSF |  |
|  | Female  (38) | 13.2 to 16.4 | 24  (16.0 to 33.8) |  | 1.1369 - 0.0598 * log SSF |  |
| Slaughter et al. ^(9)^ | Male  (50) | 9.8 | 20.4 to 8.2 | Mixed  (USA) |  | 1.21 * SSF2 - 0.008 * (SSF2)^2^ - 1-7 |
|  | Female  (16) | 10 | 24.4 to 6.8 |  |  | 1.33 * SSF2 - 0.013 * (SSF2)^2^ – 2.5 |
| Johnston et al. ^(30)^ | Male  (140) | 8 to14 | 16.5 ± 7.9 | Caucasian  (Canada) | 1.166 - 0.070 * log SSF |  |
|  | Female  (168) |  | 22.6 ± 7.5 |  | 1.144 - 0.060 * log SSF |  |
| Wendel et al. ^(27)^ | Male  (401) | 8.2 to 14.2 | 26.8  (22 to 31.9) | Mixed (Non-African)  (USA) |  | 12.74 * log SSF − 21.47 * ln Height + 87.82 |
|  | Female  (434) |  |  |  |  | 13.99 * log SSF − 21.42 * ln Height + 85.65 |
| Brook^(31)^ | Male  (12) | 1 to11 | 23.7  (13.8 to 36.5) | Caucasian  (UK) | 1.1690 - 0.0788 * log SSF |  |
|  | Female  (11) |  | 33.1  (23.5 to 38.0) |  | 1.2063 - 0.0630 * log SSF |  |
| Deurenberg et al. ^(29)^ | Male  (114) | 7 to 20 | 11 | Caucasian  (Holland) | 1.1133 – 0.0561 * log SSF + 1.7 * Age *10^-3^ |  |
|  | Female  (98) |  | 10.5 |  | 1.1187 – 0.0630 * log SSF + 1.9 * Age *10^-3^ |  |
| Alkutbe et al.^(50)^ | Male  (467) | 9 to 11 | 27.7  (5.2 – 49.4) | Saudi Arabian |  | -1.0116*Age-0.1564*Weight+0.09892*Height+q.7353*BMI-8.1772^b^ |
|  | Female  (756) | 8 to 11 | 27.2  (3 – 51.4) |  |  | -0.2069*Age+0.05958*Weight-0.03893*Height+1.4478*BMI+1.8192^b^ |

Abbreviations: SSF, sum of 4 skin-folds (biceps, triceps, sub-scapular, suprailiac); SSF2, sum of 2 skin-folds (biceps, sub-scapular); BMI, body mass index (kg/m^2^). ^a^%BF calculated from body density as %BF= [562-4-2 (Age-2)]/BD-[525-4-7 (Age-2)] ^(32)^;^b^recalculated from data provided by Alkutbe et al.^(50)^

**Supplementary Table 2 Published prediction equations for body composition in children aged 5 – 18 years**

| **Reference** | **Prediction equation** | **Population** | **Number** | **Age (y)** | **SEE (kg)** | **R^2^** |
| --- | --- | --- | --- | --- | --- | --- |
| **Published general bioimpedance spectroscopy predictions for body composition** | | | | | | |
| Moissl et al. ^(51)^ | Mixture theory equations for TBW adjusted from BMI using  K_B_ = 4.3  Db = 1.05 (kg/L)  Male ρ_ICW_ = 273.9 (ohm.cm), ρ_ECW_ = 40.5 (ohm.cm)  Female ρ_ICW_ = 264.9 (ohm.cm), ρ_ECW_ = 39.0 (ohm.cm) | USA (Caucasian, Hispanic, African-American) Germany, Sweden | 76 M,  76 F | 43.7 ± 12.4 |  |  |
| Moon et al. ^(22)^ | Mixture theory equations  K_B_ = 4.3 or 4.0*  Db = 1.05 (kg/L)  Male ρ_ICW_ = 937.2 (ohm.cm), ρ_ECW_ = 273.9 (ohm.cm)  Female ρ_ICW_ = 894.2 (ohm.cm), ρ_ECW_ = 235.5 (ohm.cm) | USA (Caucasian) | 15 M,  14 F | 18 - 27 |  |  |
| Ward et al. ^(23)^ | Mixture theory equations  K_B_ = 4.3 or 4.0*  Db = 1.05 (kg/L)  Male ρ_ICW_ = 1029.0 (ohm.cm), ρ_ECW_ = 355.5 (ohm.cm)  Female ρ_ICW_ = 927.5 (ohm.cm), ρ_ECW_ = 289.6 (ohm.cm) | Australian  (predominantly Caucasian) | 85 M,  66 F | 18 - 65 |  |  |
| **Published SFBIA prediction equations for body composition in children aged 5 – 18 years** | | | | | | |
| Rush et al. ^(34)^ | FFM = 0.622 (RI) + 0.234 (W) + 1.166 | New Zealand Caucasian, Maori, Pacific Islands | 83 M,  89 F | 5 - 14 | 2.44 | 0.96 |
| Jemaa et al. ^(35)^ | TBW = 0.083 (RI) + 0.274 (A) + 0.637 (S) + 0.310 (W) + 2.456 | Tunisian | 67 both sexes | 8 - 11 | 1.34 | 0.92 |
| De Lorenzo et al. ^(36)^ | FFM = 0.588 (RI) + 0.21 (W) + 2.33 | Italian Caucasian |  | 7.7 - 13 | 1.0 | 0.96 |
| El Harchaoui et al. ^(37)^ | TBW = 0.292 (RI) +0.221 (W) + 0.824 (S) + 0.269 | Moroccan | 82 M,  41 F | 8 - 11 | 1.51 | 0.91 |
| Horlick et al. ^(38)^ | TBW = 0.475 (RI) + 0.14 (W) + 0.725 | USA (White, African-American, Asian, Hispanic) | 664 M, 627 F | 4-18 | 0.001 | 0.95 |

SEE = standard error of the estimate; R^2^ = coefficient of determination; A = age (years); RI = resistance index (H^^2^/R50, cm^^2^/ohm); Xc = reactance at 50 kHz; (ohm) W = weight (kg); H = height (cm); S = sex (male = 1, female = 0);* K value calculated in this study for children
